# Supplementary material for: Prognostic factors for persistent obstructive symptoms in patients with Hirschsprung disease following pull-through
Source: PLoS One. 2023 Sep 8;18(9):e0290430. doi: 10.1371/journal.pone.0290430 (PMC10490837; doi:10.1371/journal.pone.0290430)
Supplement: Attachment — Submitted filename: ResponseToReviewer2_Naisya.edited.docx [file pone.0290430.s003.docx]

**RESPONSES TO REVIEWER AND EDITOR:**

The editor and reviewer comments are in italics in the material below, and our responses are regular.

***Reviewers' comments:***

*Reviewer's Responses to Questions*

*Comments to the Author*

*1. If the authors have adequately addressed your comments raised in a previous round of review and you feel that this manuscript is now acceptable for publication, you may indicate that here to bypass the “Comments to the Author” section, enter your conflict of interest statement in the “Confidential to Editor” section, and submit your "Accept" recommendation.*

*Reviewer #1: All comments have been addressed*

*Reviewer #2: All comments have been addressed*

Thank you very much for these encouraging comments from these reviewers.

*2. Is the manuscript technically sound, and do the data support the conclusions?*

*The manuscript must describe a technically sound piece of scientific research with data that supports the conclusions. Experiments must have been conducted rigorously, with appropriate controls, replication, and sample sizes. The conclusions must be drawn appropriately based on the data presented.*

*Reviewer #1: Yes*

*Reviewer #2: Yes*

Thank you very much for these encouraging comments from these reviewers.

*3. Has the statistical analysis been performed appropriately and rigorously?*

*Reviewer #1: Yes*

*Reviewer #2: Yes*

Thank you very much for these encouraging comments from these reviewers.

*4. Have the authors made all data underlying the findings in their manuscript fully available?*

*The PLOS Data policy requires authors to make all data underlying the findings described in their manuscript fully available without restriction, with rare exception (please refer to the Data Availability Statement in the manuscript PDF file). The data should be provided as part of the manuscript or its supporting information, or deposited to a public repository. For example, in addition to summary statistics, the data points behind means, medians and variance measures should be available. If there are restrictions on publicly sharing data—e.g. participant privacy or use of data from a third party—those must be specified.*

*Reviewer #1: Yes*

*Reviewer #2: Yes*

Thank you very much for these encouraging comments from these reviewers.

*5. Is the manuscript presented in an intelligible fashion and written in standard English?*

*PLOS ONE does not copyedit accepted manuscripts, so the language in submitted articles must be clear, correct, and unambiguous. Any typographical or grammatical errors should be corrected at revision, so please note any specific errors here.*

*Reviewer #1: Yes*

*Reviewer #2: Yes*

Thank you very much for these encouraging comments from these reviewers.

*6. Review Comments to the Author*

*Please use the space provided to explain your answers to the questions above. You may also include additional comments for the author, including concerns about dual publication, research ethics, or publication ethics. (Please upload your review as an attachment if it exceeds 20,000 characters)*

*Reviewer #1: The authors present their single institution experience between 2017-2022 of patients post pull through dealing with obstructive symptoms in a developing country. They evaluated 25 patients (or 22% of the 114) to determine factors for obstructive symptoms.*

*The authors need to rewrite the number of patients so that there is consistent way of # of patients and percentage in all parts of the manuscript.*

We have now rewritten the number of patients so that there is a consistent way of # of patients and percentage in all parts of the manuscript.

*I still do not understand how obstructive symptoms were evaluated or why an NGT tube is required postoperatively.*

We have now revised the Methods section: “After admission, the patients with persistent obstructive symptoms were managed with antibiotics, irrigations, and nothing per oral. They were evaluated using the following diagnostic approach: history, abdominal X-ray, contrast enema, and examination under anesthesia.^3,4^ Treatment of patients was determined according to the etiology of the obstructive symptoms, anatomic, pathological, or functional.^3,4^”

The placement of NGT was our protocol from the anesthesiologist during the surgery. The NGT was removed soon after the surgery.

We have now revised the Methods section:

“Post pull-through management

The nasogastric tube was removed soon after surgery finished.”

*They have overall provided answers to the reviewers criticisms.*

Thank you very much for these encouraging comments from this reviewer.

*Reviewer #2: (No Response)*

*7. PLOS authors have the option to publish the peer review history of their article (what does this mean?). If published, this will include your full peer review and any attached files.*

*If you choose “no”, your identity will remain anonymous but your review may still be made public.*

*Do you want your identity to be public for this peer review? For information about this choice, including consent withdrawal, please see our Privacy Policy.*

*Reviewer #1: No*

*Reviewer #2: No*
